# The Neuroprotective Effects of Alpha-Tocopherol as an Anti-Inflammatory Agent: Mechanistic Insights and Therapeutic Challenges

**DOI:** 10.3390/nu18040676

**Published:** 2026-02-19

**Authors:** Megumi H Seese, Yuanzhong Xu

**Affiliations:** 1 Brown Foundation Institute of Molecular Medicine, McGovern Medical School, University of Texas Health Science Center at Houston (UTHealth), Houston, TX 77030, USA; megumi.h.seese@uth.tmc.edu; 2 MD Anderson Cancer Center, UTHealth Houston Graduate School for Biomedical Sciences, Houston, TX 77030, USA

**Keywords:** alpha-tocopherol, vitamin E, neuroinflammation, brain health

## Abstract

Vitamin E (alpha-tocopherol, αToc), an antioxidant fat-soluble vitamin that prevents lipid peroxidation and modulates pro-inflammatory cytokine production, is well-known to be critical for neuroprotection. However, the underlying mechanism remains poorly understood. Accumulating evidence demonstrates that αToc influences neuroinflammatory processes. Here, we review recent research progress on the effects of αToc on neuroinflammation. Preclinical studies included in this narrative review were identified through PubMed searches using the keywords “alpha-tocopherol AND neuroinflammation or alpha-tocopherol AND brain AND inflammation”. While many studies support a neuroprotective role of αToc, the magnitude and direction of its effects vary substantially depending on dosage, route of administration, types of αToc substance, sex, and experimental design. The conclusions of this review apply only to αToc and should not be extrapolated to other vitamin E isoforms. Collectively, these findings suggest that αToc exerts beneficial effects primarily under specific conditions, and that a deeper understanding of the neural mechanisms mediating its neuroprotective actions may facilitate the optimization of therapeutic strategies for neuroinflammatory diseases.

## 1. Introduction

Vitamin E was identified as a critical factor for reproductive function in rats by Bishop and Evans in 1922 [1], and its relationship to health and diseases has been extensively studied for the last century. Vitamin E includes 8 isomers, which are α-, γ-, ε-, and δ-tocopherols and tocotrienols. The definition “vitamin E” remains debated [2,3,4], particularly regarding whether only α-tocopherol (αToc) should be designated as vitamin E, given that it is the most abundant and bioavailable form in the body. In this review, αToc is used to represent vitamin E, although all stereoisomers were included due to the frequent lack of specification of αToc sources in the included studies.

Oxidative stress and inflammation are associated with many abnormalities, including lipid peroxidation, DNA and protein damage, and neurodegeneration. As an antioxidant lipophilic essential nutrient, vitamin E prevents lipid peroxidation in the brain by scavenging free radicals derived from polyunsaturated fatty acids (PUFAs) within cell membranes [5,6]. Particularly, protection of PUFAs is critical for the integrity of cell membranes. The brain exhibits a high rate of O_2_ consumption due to its high mitochondrial ATP demand [7] and is a lipid-rich tissue. Thus, the antioxidant property of αToc is essential for neuroprotection.

Furthermore, αToc has been shown to reduce markers of inflammatory responses through the inhibition of protein kinase C (PKC) [8] or via antioxidant pathways [9]. Godbout et al. demonstrated that αToc supplementation attenuated peroxide radical production and interleukin (IL)-6 levels following lipopolysaccharide (LPS) exposure in primary microglia and in mouse brains [10]. Several studies have also studied the effects of αToc on inflammatory and oxidative stress responses, as well as mouse social behavior changes, in mouse models [11,12,13,14,15]. Collectively, these findings provide strong evidence that αToc status influences neuroinflammation, although reported effects vary considerably across experimental designs.

While the effects of αToc as an anti-inflammatory property have been extensively investigated, the mechanism by which αToc prevents or modulates neuroinflammation remain incompletely understood. Accordingly, the central question addressed in this narrative review is which experimental models have been used to examine the effects of αToc on neuroinflammation. To this end, we summarize and evaluate preclinical in vivo and in vitro studies assessing the impact of αToc on neuroinflammatory processes. This review aims to provide an up-to-date synthesis of current evidence regarding the mechanistic insights and therapeutic challenges of αToc in modulating neuroinflammation.

## 2. Preclinical Studies Selection Criteria

**Figure 1:**
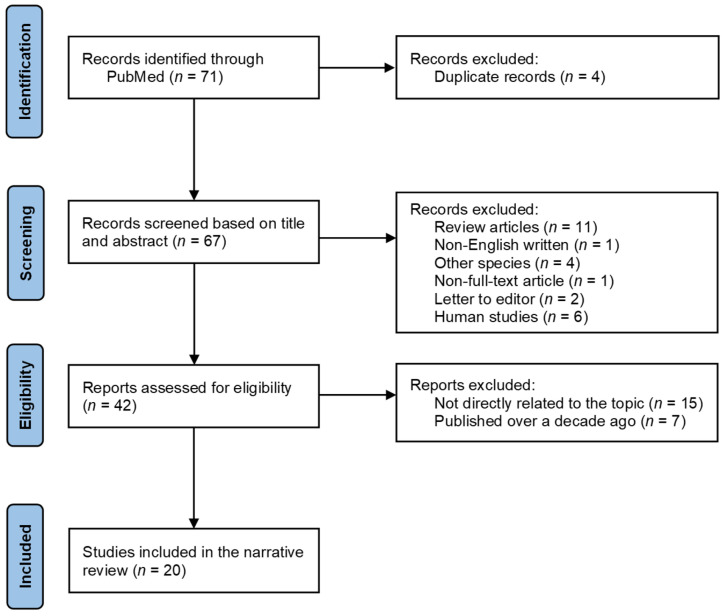
Flow diagram for study selection. This narrative review included preclinical studies that were searched using the keywords “alpha-tocopherol AND neuroinflammation” or “alpha-tocopherol AND brain AND inflammation” through PubMed and published within the last 10 years. Exclusion criteria included studies that were not related to αToc or neuroinflammation; studies examining other vitamin E forms (e.g., γToc and tocotrienols); studies not written in English; studies without full-text access; and studies conducted in humans or non-rodent species (e.g., horses and chickens).

The identification of preclinical studies included in this narrative review followed a process similar to that of a systematic literature process (Figure 1). Searches were conducted in the PubMed database using the following keywords with the free full-text filter applied: “alpha-tocopherol AND neuroinflammation” or “alpha-tocopherol AND brain AND inflammation”. A total of 41 and 30 articles, respectively, were initially identified and screened based on their titles and abstracts. When the title and abstract were insufficient for study selection, additional sections of the articles were reviewed. Exclusion criteria included studies that were not related to αToc or neuroinflammation; studies examining other tocopherol isomers (e.g., γ-tocopherol (γToc)) or other forms of vitamin E (e.g., tocotrienols); studies not written in English; studies without full-text access; and studies conducted in humans or non-rodent species (e.g., horses and chickens). To minimize variability in vitamin E terminology, we excluded studies in which different tocopherol isomers were grouped together. Based on the PubMed search, the effects of αToc on neuroinflammation have been investigated for more than 30 years. To emphasize recent preclinical research, only studies published within the past 10 years were included in a summary table. From publications between 2015 and 2025, six in vitro and 15 in vivo studies were identified, including one study that incorporated both in vitro and in vivo approaches.

## 3. The Effects of αToc on Neuroinflammatory Response

### 3.1. The Distribution of αToc in the Brain

Vitamin E can cross the blood–brain barrier via scavenger receptor class B type I, α-tocopherol transfer protein (αTTP), and ATP-binding cassette A and G [16], supporting the distribution and bioavailability of αToc in the brain. Specifically, αTTP plays a crucial role in packaging vitamin E into lipoproteins, thereby facilitating its transport into the plasma for subsequent uptake by extrahepatic tissues. Accordingly, αTTP is essential for maintaining vitamin E homeostasis by regulating the turnover and tissue delivery of αToc. Importantly, αTTP is expressed in several brain regions, including the cerebellum, cerebellar cortex, and hippocampus [14,17]. This regional localization may regulate αToc turnover by protecting it from degradation and enabling targeted delivery to specific cell types, such as astrocytes. Consistent with this notion, αToc turnover occurs more rapidly in serum, liver, and peripheral tissues (e.g., heart and adipose tissue), whereas depletion of αToc in the brain is markedly slower [18,19]. After approximately 50 weeks of αToc depletion in rats, neurological tissues still retained ~5% of their initial αToc concentration, whereas αToc levels in other tissues became undetectable within 16 weeks [18]. Within the brain, αToc turnover rates vary by region, with the cerebellum exhibiting faster turnover than other brain regions [20]. Moreover, recent evidence indicates that αTTP is selectively expressed in cerebellar astrocytes rather than neurons, suggesting an essential role for αTTP in brain function [21]. Together, the regional and cell-type-specific expression of αTTP, along with differential αToc turnover patterns, provides important insight into the tissue- and cell-specific roles of αToc in maintaining neural homeostasis.

### 3.2. The Importance of αToc Status in Immune System

αToc plays a critical role in regulating the immune system and inflammation, including cytokine production and immune cell activities. Hatam and Kayden reported that immune cells contain substantially higher concentrations of αToc than red blood cells [22], suggesting an important role for αToc in immune function. In vivo, αToc supplementation promotes lymphocyte proliferation, helper T-cell activity, and IL-2 production, and enhances overall immune responses, including increased natural killer cell activity and macrophages’ phagocytic ability in the lungs [23]. Supporting these observations, Finno et al. (2019) reported that αTTP knockout (*Ttpa^−/−^*) mice fed an αToc-deficient diet (<10 mg synthetic α-Toc acetate/kg) for 6 months exhibited elevated oxidative stress and the upregulated expression of genes associated with innate immune responses [24]. Collectively, these findings indicate that αToc status is a key determinant of immune system function.

### 3.3. The Anti-Inflammatory Role of αToc In Vitro

Recent studies have examined the effects of αToc on inflammation and oxidative stress in microglia cells [25,26,27], reflecting their central role in immune surveillance within the brain, as well as in neurons, astrocytes [28], and oligodendrocytes [29]. In murine microglia cells, αToc supplementation attenuated LPS-induced neuroinflammation [25]. Furthermore, combined treatment with phospholipids and αToc reduced oxidative stress and inflammation in a murine microglial cell line. This combination enhanced cell viability in activated microglia by decreasing nitric oxide and tumor necrosis factor-alpha (TNF-α) while increasing transforming growth factor beta 1 expression [27]. In contrast, microglia cells treated with triglyceride-rich lipoproteins and αToc showed only partial and non-significant prevention against neuroinflammation [26]. Although direct inflammatory markers were not measured in neurons, astrocytes, or oligodendrocytes [28,29], αToc treatment consistently mitigated neurodegenerative processes in these cell types, including oxidative stress and cell death. Torres et al. further demonstrated a synergistic effect of αToc and docosahexaenoic acid on cellular function in motor neurons from the lumbar spinal cord of neonatal rats exposed to chronic excitotoxicity, showing that combined nutrient treatment rescued the cell viability compared with untreated controls [30]. Collectively, these findings indicate that αToc, either alone or in combination with other nutrients or bioactive compounds, significantly reduces pro-inflammatory cytokine production and improves cellular function in microglia cells, supporting a meaningful role for αToc in neuroimmune regulation.

### 3.4. The Regulatory Role of αToc on Neuroinflammation and Neuroprotection In Vivo

Preclinical studies have extensively examined the role of αToc in modulating neuroinflammation, oxidative stress, and neurological function across a wide range of experimental study designs. A summary of recent studies is shown in Table 1. Collectively, these studies suggest that αToc may exert neuroprotective effects, although efforts vary depending on experimental study designs, dosage/formulation/administration of αToc, disease context, and interaction with other nutrients or bioactive compounds.

#### 3.4.1. αToc on Systemic Inflammation

Models of systemic inflammation and αToc deficiency provide mixed evidence regarding the role of αToc in modulating LPS-induced neuroinflammation. Recent αToc-deficiency studies using *Ttpa^−/−^* mice demonstrated that LPS-induced systemic inflammation triggered neuroinflammation at 4 and 24 h post-injection in the hippocampus of both C57BL/6J (wild type, WT) and *Ttpa^−/−^* mice. However, these studies did not detect significant effects of αToc deficiency on neuroinflammation or oxidative stress [31], suggesting that the acute inflammatory challenge induced by LPS administration may have been sufficiently robust to mask genotype-specific differences. Notably, nine weeks of αToc-depletion revealed an effect of αToc deficiency on murine grip strength following intraperitoneal (i.p.) LPS injection, indicating that αToc status may influence muscle function under systemic inflammatory conditions [32], even though the expression of inflammatory markers was not altered by genotype. These findings, however, are subject to several limitations, including the absence of WT mice maintained on an αToc-sufficient diet and the exclusive use of male animals, which constrain interpretation.

#### 3.4.2. αToc on Chemical-Exposed Inflammation

Evidence from the in vivo chemical-exposed inflammation model, such as acrylamide exposure and pyrethroid-induced toxicity, is less supportive of a direct neuroprotective role for αToc. αToc administration showed limited efficacy in attenuating pyrethroid-induced tissue damage [36]. In contrast, αToc treatment improved vapor exposure-induced kidney and lung damage associated with liquid formulations, whereas ascorbic acid administration accounted for most of the observed tissue recovery. In chicken embryos but not rodent models, similarly, αToc treatment did not reduce acrylamide- or dioxins-induced oxidative stress markers, such as malondialdehyde (MDA) and cyclooxygenase-2, in the cerebrum, cerebellum, medulla oblongata [45], or in the whole brain [46]. Overall, evidence from chemical-induced inflammation or oxidative stress models suggests that αToc confers limited neuroprotection.

#### 3.4.3. αToc on Chronic Inflammation and Neurodegenerative Disease

Accumulating evidence demonstrates that αToc exerts consistent effects of neuroprotection across a range of animal models of chronic inflammation and neurodegenerative disease. In rodent models of arthritis [34], Parkinson’s disease [33], traumatic brain injury [37], stroke [42], Alzheimer’s disease [28,41], epilepsy [39,44,47], and chronic alcohol exposure [35], αToc administration, either alone or in combination with pharmacological agents such as doxycycline, etodolac, or lovastatin, frequently attenuated oxidative stress and neuroinflammatory markers in the brain while improving behavioral and cognitive outcomes.

Notably, in contrast to many studies highlighting the benefits of αToc supplementation, one report demonstrated that excessive αToc supplementation increased brain αToc concentrations and exacerbated stroke-induced microglial activation, oxidative stress, neurodegeneration, and lesion volume in WT mice [43]. Although these findings raise concerns regarding the potential risks of high-dose αToc intake in the context of stroke, the study did not establish whether brain αToc levels reached toxic thresholds, leaving uncertainty as to whether the observed effects reflect direct toxicity or region-dependent differences in αToc distribution.

#### 3.4.4. Synergistic Effects of αToc with Other Nutrients

Some evidence suggests that αToc may exert synergistic neuroprotective effects when combined with other nutrients, such as vitamin C or PUFAs. For example, a mouse model of combined vitamin C and αToc deficiency revealed impairments in conditioned fear-associated memory [38], potentially due to increased neuroinflammation in the hippocampus. In a stroke model, a parental nutrition formulation containing omega-3 fatty acids along with αToc showed significantly attenuated neuroinflammation, improved mitochondrial function, and reduced infarct size and stroke severity, while αToc supplementation alone was less effective [40]. These findings underscore the importance of evaluating αToc’s synergistic interactions with other nutrients in neuroprotection.

Overall, preclinical evidence supports a conditional, but not universal, neuroprotective role for αToc, primarily mediated through the modulation of oxidative stress and inflammatory pathways. Nevertheless, key gaps remain, including the frequent use of single-sex models, inconsistent quantification of brain αToc concentrations, and the lack of appropriately designed studies specifically examining the regulatory role of αToc in neuroinflammation.

## 4. Discussion

The purpose of this review was to summarize recent key findings on the effects of α-tocopherol (αToc) on neuroinflammation. The studies synthesized in this review highlight the complex and context-dependent role of αToc in modulating neuroinflammation and oxidative stress across diverse preclinical models. Although many investigations support a neuroprotective role for αToc, the magnitude and direction of its effects vary substantially depending on dosage, route of administration, αToc formulation, sex, and experimental design. Collectively, these findings suggest that αToc exerts beneficial effects primarily under specific conditions, whereas acute or excessive exposure may attenuate or even counteract its protective capacity.

Among studies focusing on microglia, αToc generally attenuated pro-inflammatory cytokine production, although the magnitude of effect varied depending on the experimental design. For instance, La Torre et al. reported a significant reduction in LPS-induced neuroinflammation following αToc supplementation, supporting its role as an anti-inflammatory modulator [25]. In contrast, Espinosa et al. observed only partial and non-significant protection in microglia cells treated with triglyceride-rich lipoproteins [26]. Previous literature also showed that αToc had potential effects on microglia activation and anti-inflammatory properties throughout the modulation of p38 mitogen-activated protein kinase and NFκB activation in vitro [48], indicating potential effects of microglial metabolic reprogramming. This variability underscores the importance of considering microglial phenotype and metabolic state when interpreting the immunomodulatory potential of αToc.

Consistent with its anti-inflammatory property, either oral or i.p. administration of αToc reduced the production of several pro-inflammatory cytokines, including TNF-α, IL-1β, and IL-6, in the brain [33,35,37,41,44]. Chronic ethanol consumption elevated pro-inflammatory cytokine levels while reducing anti-inflammatory markers; these effects were attenuated by αToc administration in a dose-dependent manner [35]. However, not all inflammatory markers were responsive to αToc. In some contexts, αToc alone did not produce significant effects or only partially improved oxidative stress, such as in the brain of chicken embryos [45]. In addition, studies using lipid emulsions rich in omega-3 fatty acids demonstrated that αToc alone was less effective at reducing stroke-induced neurodegeneration, despite its critical role in preventing PUFA oxidation [40]. Across many studies, substance-induced inflammation was attenuated by αToc supplementation, either alone or synergistically with bioactive compounds. Together, these observations highlight the importance of nutrient interactions rather than single-nutrient supplementation. These findings suggest that the anti-inflammatory efficacy of αToc is highly dependent on the nature of the inflammatory trigger and specific physiological contexts, including infection, elevated oxidative stress, or chemical exposure.

Across the studies included in this review, several reports demonstrated that oral or i.p. αToc, administered alone or in combination with other compounds, consistently reduced oxidative stress in murine brains. These effects were evidenced by directly scavenging oxidants, as indicated by decreased levels of MDA and nitrites [28,33,37,42,44] and/or by enhancement of antioxidant enzymes, such as SOD and catalase [28,34,41]. These findings align with prior reports [10,11,13,14] and support a regulatory role for αToc in redox homeostasis, potentially through the modulation of redox-sensitive transcription factors [49]. In addition, LPS-induced pro-inflammatory cytokine production was attenuated by αToc treatment in mice [10], suggesting interference with TLR-mediated signaling and downstream NF-κB activation [50]. Consistently, αToc treatment also reduced inflammatory responses in the microglial cell line, further supporting its role in regulating neuroinflammation. Although studies directly examining protein kinase C (PKC) signaling were not included in this review, previous evidence indicates that αToc modulates PKC activity [51], particularly PKCα activation [52]. Elucidating these mechanistic pathways will be critical for defining how αToc exerts its anti-inflammatory effects in the central nervous system.

Multiple studies have reported that αToc improves behavioral outcomes, including locomotion, grip strength, cognition, anxiety-like behavior, and seizure susceptibility. In epilepsy models, repeated αToc treatment attenuated kainic acid-induced astrogliosis, microglial activation, oxidative damage, and synaptic dysfunction while also reducing neuronal network hyperexcitability in the hippocampus [39,44]. Similarly, traumatic brain injury (TBI) and Parkinson’s disease-like models demonstrated improvements in motor and cognitive function, particularly when αToc was administered at higher doses or in combination with anti-inflammatory agents such as doxycycline [33,37]. However, these behavioral benefits were not universal. For instance, combined vitamin C and αToc deficiency models did not show improvements in most behavioral outcomes following αToc supplementation [38]. Collectively, these findings suggest that specific behavioral domains may be more sensitive to αToc-related antioxidant insufficiency, particularly those involving hippocampal-dependent associative memory.

While most studies have focused on the effects of αToc supplementation, reports showing that excessive αToc intake can increase stroke-induced neuroinflammation and lesion volume in experimental animals raise concerns about excessive αToc accumulation in the brain (25 nmol/g tissue in WT mice) [43]. Notably, the oral dose used in that study (150 IU dl-αToc/kg diet = 67.5 mg/kg diet) was comparable to or lower than doses used in other studies and in AIN-93G or M diets [53]. Interestingly, their brain αToc accumulation was not significantly higher than the other studies (~25–30 nmol/g in WT mice fed with 75 mg for 40 weeks or 600 mg dl αToc/kg for 24 weeks) [54,55]. Intraperitoneal dl-αToc administration (400 IU dl-αToc/kg BW daily) is likely to result in supraphysiological and rapid tissue accumulation compared to with oral administration [56], which would cause αToc toxicity. Collectively, these findings suggest that αToc may exacerbate pathology under specific conditions; however, the adverse effects of excessive vitamin E status on neuroinflammation remain poorly defined, in contrast to the well-documented effects of vitamin E deficiency or adequacy in attenuating neuroinflammation [32,38] or improving neurological outcomes [13,14], respectively. It is challenging to deplete αToc accumulation in the brain of WT mice, even after 12 weeks αToc depletion period (~15 nmol/g tissue) [31]. Identifying the appropriate dose of αToc for vitamin E status (deficiency, adequacy, and excess) is critical for neuroprotective research by the nutrient.

All studies included in this review used animals of a single sex, predominantly male rodents, despite evidence suggesting sex differences in αToc levels and behavior [17,57]. Several studies have reported that αToc concentrations are higher in females than in males in both rodents [17,58] and humans [59], indicating sex-specific differences in αToc bioavailability. In mice, αToc levels in the cerebellum are lower than in the other brain regions, including the cortex, hippocampus, brainstem, and midbrain [17], highlighting regional differences in brain αToc distribution and suggesting potential implications for motor coordination. Notably, sex differences in inflammatory responses have been documented in murine brains [60]. However, studies directly examining the sex-specific effects of αToc on neuroinflammation and behavioral outcomes remain limited. Addressing this gap will be critical for advancing mechanistic and translational research on vitamin E metabolism and its anti-inflammatory effects in the brain.

Several limitations of this review should be acknowledged. First, because this review focused exclusively on recent studies, relatively few studies have directly examined the effects of αToc alone, which may introduce selection bias. In addition, the exclusion of studies examining other forms of vitamin E, such as γ-tocopherol (γ-Toc) and tocotrienols, represents a limitation. Several studies have reported similar or distinct effects of these vitamin E isomers on inflammatory responses compared to αToc [61,62,63,64,65], which may influence the interpretation of conclusions drawn exclusively from αToc-focused studies. Nevertheless, some studies have shown limited biological effects of γ-Toc in human aortic cells [66] or in mice [67], likely due to the relatively lower bioavailability of these vitamin E isomers compared to αToc [68]. Moreover, given that αToc is the predominant biologically active form of vitamin E and is used to define human vitamin E requirements, this narrative review focused solely on αToc. A brief comparison of key differences among αToc, γToc, and tocotrienols is summarized in Table 2. However, accumulating evidence supporting the biological relevance of other vitamin E forms, together with ongoing debate regarding vitamin E nomenclature, highlights the need for more comprehensive investigations in future studies.

Second, substantial heterogeneity existed across studies with respect to dosage and routes of administration (e.g., intraperitoneal bolus, intravenous injections, oral administration via diet or drinking water, and oral gavage) and in methods used for inflammation induction (e.g., LPS, kainic acid, rotenone, or injury). These methodological differences likely affect αToc biodistribution and bioavailability, as well as the magnitude and nature of neuroinflammatory responses. Furthermore, inconsistent reporting of statistical analysis and unclear comparison strategies further complicate cross-study interpretation. Third, relative few studies quantified the brain and serum αToc concentrations, which hinder mechanistic interpretation and the linkage between αToc exposure and functional outcomes. Finally, translational challenges remain due to interspecies differences in αToc metabolism, difficulties in attaining brain-relevant concentrations in humans, and inconsistency among clinical studies. Compared with tightly controlled animal models, clinical studies involve substantially more complex and variable factors that may obscure potential neuroprotective effects.

## 5. Conclusions and Perspectives

**Figure 2:**
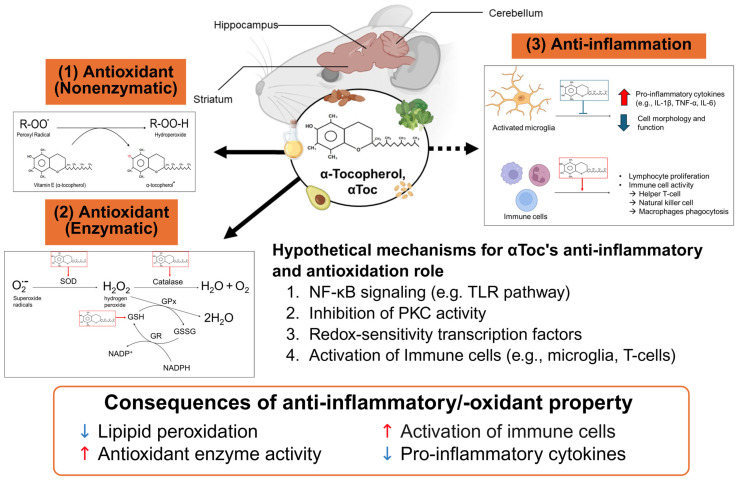
Schematic diagram illustrating the neuroprotective actions of vitamin E in the murine brain. α-Tocopherol (αToc) is mainly found in oil, seeds, nuts, and green leafy vegetables. As an anti-inflammation and antioxidant nutrient, αToc attenuates lipid peroxidation and reduces neuroinflammation in brain regions such as the striatum, cerebellum, and hippocampus, either through the (1) direct scavenging of free radicals, (2) enhancement of endogenous antioxidant pathway, and/or (3) suppression of cytokine production. Solid arrows show mechanisms that are well supported by experimental evidence, while the dashed line indicates those that remain more hypothetical. Red arrows indicate increased direction while blue arrows mean decreased conditions. This figure was created in https://BioRender.com. Abbreviations: GPx, glutathione peroxidase; GSH, glutathione; GSSG, glutathione disulfide; GR, glutathione reductase; IL, interleukin; MDA, malondialdehyde; NO, nitric oxide; PKC, protein kinase C; SOD, superoxide dismutase; TLR, toll-like receptor; TNF-α, tumor necrosis factor-α.

The conclusions of this review apply only to αToc and should not be extrapolated to other vitamin E isoforms. The observed neuroprotective effects of αToc may be mediated by (1) direct scavenging of free radicals, (2) enhancement of endogenous antioxidant pathway, and/or (3) suppression of cytokine production (Figure 2). However, the effects of αToc per se remain incompletely understood, as its efficacy is highly dependent on dose, disease context, administration route, and certain environments, such as increased systemic inflammation/oxidative stress circumstances. Although αToc modulates biomarkers associated with neuroinflammation in the brain, careful consideration of study design and physiological relevance is essential to avoid overlooking potential adverse neurological effects.

Future studies should incorporate both sexes, standardized inflammatory and oxidative biomarkers, and dose–response designs that encompass deficiency, sufficiency, and excess. In particular, mechanistic studies are needed to elucidate how αToc attenuates oxidative stress and inflammation within specific brain regions under different pathological contexts. Furthermore, assessment of tissue αToc distribution in conjunction with functional outcomes will be critical for translating preclinical findings into evidence-based nutritional or therapeutic recommendations.

## Figures and Tables

**Table 1:** Summary of αToc supplementation on neuroinflammation: Preclinical outcomes.

Author	Study Design	Route of Administration (Dosage)	Targeted Brain Region	Summary
Seese et al. (2024) [31]	Male WT and *Ttpa^−/−^* mice with an αToc-deficient diet for 12 weeks ± single LPS i.p. injection.	Oral (0 mg/kg diet)	Hippocampus	Systemic LPS administration caused increased neuroinflammation and decreased grip strength. The impact of αToc deficiency did not affect LPS-induced inflammatory and oxidative stress responses.
Hashida et al. (2024) [32]	Male WT and *Ttpa^−/−^* mice with an αToc-deficient diet for 9 weeks ± single LPS i.p. injection.	Oral (0 mg/kg diet)	Cerebellum	LPS administration caused an acute systemic and neuroinflammation and decreased grip strength, especially in *Ttpa^−/−^* mice. αToc deficiency may cause exacerbate reductions in grip strength brought on by systemic inflammation.
Singh and Chauhan (2024) [33]	Male Wistar rats (4–5 months old) with rotenone-induced Parkinson’s disease symptoms ± oral drug treatment of αToc, Doxy, αToc + Doxy, and i.p. ropinirole.	Oral (5 or 10 mg/kg)	Striatum	The synergic effect of Doxy with αToc contributed to the prevention of Parkinson’s disease-like symptoms in rotenone-injected rats by antioxidant, anti-inflammatory, as well as neuroprotective function in the striatum.
Moreira et al. (2022) [34]	Male *Holtzman* rats (12 months old) ± Freund’s adjuvant-induced polyarthritis + i.p. injection of free αToc or oral nanoparticles, free αToc, or nano-encapsulated αToc for 23 days.	i.p. or oral (100 mg/kg)	Whole brain	αToc administration (either i.p. or oral nanoparticle) reduced arthritis-induced inflammation in the muscle and oxidative stress in the brain, suggesting the effects of αToc on systemic inflammation and oxidative stress.
Villas Boas et al. (2022) [35]	Male Wistar rats (45–60 days old) with ethanol exposure ± oral gavage of αToc.	Oral gavage (100, 200, or 300 mg/kg in corn oil)	Amygdala Medial hypothalamic nucleus	αToc treatment attenuated ethanol-induced anxious behavior and decreased cytokine production in amygdaloid and medial hypothalamic nucleus as anxiolytic and anti-inflammatory activity. The pharmacological effects of αToc are dose dependent.
Al-Omar et al. (2020) [36]	Male albino mice (3–4 months old) with pyrethroids exposure ± ascorbic acids or αToc in water.	Oral (100 mg/kg)	Cerebrum Diencephalon	Pyrethroid-induced tissue damage and toxicities can be mitigated by ascorbic acids or αToc; however, the effectiveness varies in degree and location.
Rana et al. (2020) [37]	Male Wistar rats (4–5 months old) with TBI ± Doxy, αToc, and Doxy + αToc for 28 days.	Oral (5 or 10 mg/kg)	Striatum Cortex	The synergistic effect of Doxy & αToc may be effective in neuroprotection due to its anti-inflammatory, antioxidant, and neurotransmitter effects, and improved behavioral function.
Elfakhri et al. (2019) [28]	5XFAD female mice (4 months old) with i.p. etodolac, oral αToc, or etodolac + αToc (COMB) in water for 1 month.	Oral (10 mg/kg BW)	Hippocampus Cortex	The COMB improved the blood-brain barrier function, decreased total Aβ levels, increased synaptic markers expression, and attenuated neuroinflammation and oxidative stress both in vitro and in vivo studies.
Takahashi et al. (2019) [38]	SMP30/αTTP DKO and WT male mice (5 weeks old) ± vitamin C and αToc.	Oral (0 or 500 mg/kg diet)	Hippocampus	Vitamin C and αToc deficiency in DKO mice impaired conditioned fear memory, possibly due to increased neuroinflammation in the hippocampus.
Ambrogini et al. (2018) [39]	Adult male Sprague-Dawley albino rats with kainite-induced seizures ± i.p. bolus αToc for 15 days.	i.p. (250 mg/kg & 2 mg/kg BW)	Hippocampus	As anti-epileptogenic role, αToc treatment improved kainic acid-induced seizures by decreased neuroinflammation, miRNA expression, astrogliosis, and microglial activation in the hippocampus of rats.
Berressem et al. (2016) [40]	Stroke-induced female CD-1 mice with formulas.	i.v. (~0.2 mg/mL)	Striatum	Ω-3 fatty acids improved neurological impacts by strokes. For stabilizer of long-chain fatty acids, αToc is critical to add the lipid emulsion.
Wang et al. (2016) [41]	APPswe/PS1dE9 or WT male mice (8 mo) ± oral *RRR* αToc quinine for 4 weeks.	Oral gavage (100 mg/kg)	Hippocampus Cortex	αToc quinine treatment prevented spatial memory deficits, reduced Aβ oligomers, and inhibited neuroinflammation and oxidative stress.
Guimarães et al. (2015) [42]	Stroke-prone spontaneously hypertensive male rats (15 weeks old) ± daily orogastric gavage of synthetic αToc, lovastatin and αToc for 4 weeks.	Orogastric gavage (120 IU)	Hippocampus	αToc or lovastatin improved cognitive and memory function as well as decreased oxidative stress levels against stroke-induced neurological implications.
Khanna et al. (2015) [43]	Male *Ttpa^−/−^*, heterozygous, and WT mice (21 days old) with middle cerebral artery occlusion surgery ± αToc diets for 10 weeks.	Oral (0 or 150 IU/kg diet)	Whole brain	High αToc supplementation may cause adverse effects of stroke by increased neuroinflammation and neurodegeneration.
Ambrogini et al. (2014) [44]	Adult male Sprague-Dawley albino rats with kainite-induced seizures ± i.p. bolus αToc for 4 days.	i.p. (250 mg/kg BW)	Hippocampus	αToc treatment for kainic acid-induced status epilepticus reduced astrocytosis, microglia activation, pro-inflammatory cytokine production, neurodegeneration, and spine loss, and enhanced dendritic NFs and synaptophysin.

Abbreviations: Aβ, amyloid beta; αToc, α-tocopherol; αTTP, α-tocopherol transfer protein; BW, body weight; DKO, double-knockout; Doxy, doxycycline; i.p., intraperitoneal; i.v., intravenous; LPS, lipopolysaccharide; SMP30, senescence marker protein 30; TBI, traumatic brain injury; *Ttpa^−/−^,* αTTP knockout; WT, wild-type.

**Table 2:** Summary of the comparison of key differences among αToc, γToc, and tocotrienols.

	αToc	γToc	Tocotrienols
Structure	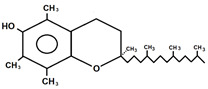 Chromanol ring (R_1_: CH_3_^−^, R_2_: CH_3_^−^) + saturated side chain	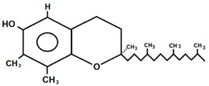 Chromanol ring (R_1_: H^−^, R_2_: CH_3_^−^) + saturated side chain	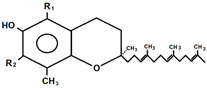 Chromanol ring (R_1_/R_2_: CH_3_^−^ and/or H^−^) + unsaturated side chain
Bioavailability	αToc >> γToc > Tocotrienols		
Antioxidant activity	α > β > γ > δ		
Anti-inflammatory mechanism	Scavenges reactive oxygen species Inhibit the activity of protein kinase C (PKC)	Scavenge reactive nitrogen species Potential inhibition of cyclooxygenase activity	Antioxidant potential

This summary was referred to throughout [69,70,71,72].

## Data Availability

No new data were created due to a narrative review article.

## Abbreviations

The following abbreviations are used in this manuscript:

αToc	alpha-tocopherol
αTTP	α-tocopherol transfer protein
IL	interleukin
i.p.	intraperitoneal
LPS	lipopolysaccharide
PUFA	polyunsaturated fatty acids
TNF-α	tumor necrosis factor-alpha
*Ttpa**^−/−^*	α-tocopherol transfer protein knockout
WT	wild type
